# How do healthcare professionals interview patients to assess suicide risk?

**DOI:** 10.1186/s12888-017-1212-7

**Published:** 2017-04-04

**Authors:** Rose McCabe, Imren Sterno, Stefan Priebe, Rebecca Barnes, Richard Byng

**Affiliations:** 1 University of Exeter Medical School, Room 1.05, College House, Exeter, EX1 2 LU UK; 2 Live Well Suffolk, 8 Turret Lane, Ipswich, IP4 1DL UK; 3 Unit for Social and Community Psychiatry, Queen Mary University of London, Newham Centre for Mental Health, London, E13 8SP UK; 4 University of Bristol, Office Room 1.05 Canynge Hall, 39 Whatley Road, Bristol, BS8 2PS UK; 5 Plymouth University Peninsula Schools of Medicine and Dentistry, N32, Tamar Science Park, Drake Circus, Plymouth, Devon PL4 8AA UK

**Keywords:** Suicide, Risk, Communication, Assessment, Conversation analysis, Mixed methods, Mental health care

## Abstract

**Background:**

There is little evidence on how professionals communicate to assess suicide risk. This study analysed how professionals interview patients about suicidal ideation in clinical practice.

**Methods:**

Three hundred nineteen video-recorded outpatient visits in U.K. secondary mental health care were screened. 83 exchanges about suicidal ideation were identified in 77 visits. A convenience sample of 6 cases in 46 primary care visits was also analysed. Depressive symptoms were assessed. Questions and responses were qualitatively analysed using conversation analysis. *χ* ^2^ tested whether questions were influenced by severity of depression or influenced patients’ responses.

**Results:**

A gateway closed question was always asked inviting a yes/no response. 75% of questions were negatively phrased, communicating an expectation of no suicidal ideation, e.g., “No thoughts of harming yourself?”. 25% were positively phrased, communicating an expectation of suicidal ideation, e.g., “Do you feel life is not worth living?”. Comparing these two question types, patients were significantly more likely to say they were *not* suicidal when the question was negatively phrased but were not more likely to say they were suicidal when positively phrased (*χ* ^2^ = 7.2, df = 1, p = 0.016). 25% patients responded with a narrative rather than a yes/no, conveying ambivalence. Here, psychiatrists tended to pursue a yes/no response. When the patient responded no to the gateway question, the psychiatrist moved on to the next topic. A similar pattern was identified in primary care.

**Conclusions:**

Psychiatrists tend to ask patients to confirm they are not suicidal using negative questions. Negatively phrased questions bias patients’ responses towards reporting no suicidal ideation.

**Electronic supplementary material:**

The online version of this article (doi:10.1186/s12888-017-1212-7) contains supplementary material, which is available to authorized users.

## Background

Almost one million people die by suicide every year worldwide, equating to one suicide every 40 s [1]. Suicide risk screening and appropriate intervention is clinically important in both secondary and primary care. Around one in four people who take their life have been in contact with mental health services the year before death in the U.K. [2] and around one in three in the U.S. [3]. The majority of depressive disorders are diagnosed and treated in primary care [4–6]: 45% of people who took their life had been seen in primary care the month before death in the U.K. [7] with a similar figure of 47% in the U.S. [3]

Communicating about suicidal ideation is a delicate activity for both clinicians and patients. Omerov et al. [8] note a widely-held belief among professionals that enquiring about suicidal ideation can increase suicidal tendencies. Cole-King and Lepping note that professionals in the U.K. may feel disinclined to enquire too deeply because of lack of confidence in knowing how to ask and how to respond [9]. From the patient’s perspective, communicating about suicidal thoughts and plans is complex. Patients may disclose suicidal thoughts, be ambivalent and not fully disclose them or may have made up their mind to attempt suicide and make every attempt to conceal this [10]. Moreover, suicidal thoughts are dynamic and can change rapidly [11]. People with experience of suicidal thoughts and attempts report that willingness to disclose distressing thoughts and plans is dependent on trust and the relationship [12, 13].

Silverman and Berman [14] suggest that assessing suicidal risk in clinical practice is influenced by the skills and philosophy of the individual clinician. Nonetheless, there are various guidelines on what to assess including life history, previous suicidal attempts and mental state [15–17], `along with helpful frameworks for how to assess risk [12, 18, 19]. There is considerably less guidance, however, on how to interview patients about suicidal ideation. This is important because *how* doctors and other professionals ask questions, i.e., the words and phrasing that they use, influences the patient’s response [20]. Some guidance recommends asking neutral or non-leading questions [21] and/or direct questions (e.g., “Have you had any thoughts about killing yourself?) [22].

A growing body of research on medical interaction has found that yes/no questions are prevalent in medical interaction and communicate an expectation in favour of either ‘yes’ or ‘no’ responses through their grammatical structure and specific words that favour ‘yes’ or ‘no’ responses [23], e.g., “Are you feeling low?” is framed positively, inviting agreement to “feeling low” [24, 25]. Conversely, “Not feeling low?” is negatively framed inviting agreement to “not feeling low”. Specific words with positive or negative polarity further reinforce bias in medical questions [26]. Words such as ‘any’, ‘ever’, ‘at all’ reinforce negative bias (e.g., “Any negative thoughts?”) while words such as ‘some’ reinforce positive bias (e.g., “Do you have some pain here?”) [26].

However, there are no observational studies of how patients are interviewed about suicidal ideation in practice. Hence, this study aimed to analyse how psychiatrists ask questions about suicidal ideation and how patients respond in community mental health care. A small convenience sample in primary care was also analysed.

## Methods

This was a mixed methods study, involving qualitative and quantitative analyses.

### Data collection

Three hundred nineteen psychiatrist-patient appointments in outpatient psychiatric clinics in urban, semi-rural and rural areas in the U.K. were audio-visually recorded. Psychiatrists and their patients meeting DSM-IV criteria for schizophrenia/schizoaffective disorder/major depressive disorder were asked to participate. Consecutively attending patients were approached by a researcher from June 2001-June 2002, March 2006-January 2008 and September 2011-October 2012. Participants were informed that the study was focusing on psychiatrist-patient communication. The patient consent rate was 45.5%.

Patients’ symptoms were assessed. Patients with schizophrenia were assessed by researchers not involved in treatment and unaware of the content of the consultation, using the Positive and Negative Syndrome Scale (PANSS) [27]. Inter-rater-reliability was good (Cohen’s kappa = 0.75). Patients with depression self-rated symptoms on the Beck Depression Inventory (BDI) [28].

Based on symptom ratings, patients were categorised as experiencing more or less suicidal ideation. More suicidal ideation was: endorsing item 9 on the BDI (thoughts of killing oneself) (patients with depression) or ≥5 (maximum score 7) on the PANSS depression item incorporating suicidal ideation (patients with schizophrenia). Less suicidal ideation was: 0 on the BDI item or <5 on the PANSS item.

In primary care, a convenience sample of 46 visits for early management of depression (≤4 weeks after diagnosis) from two practices was screened (for full details of data collection, see Karasz et al. [29]. Depression visits were identified by a Hospital Anxiety and Depression Scale score ≥8 or GP diagnosis of depression. The consent rate was 48.5%.

### Data analysis

#### Qualitative conversation analysis

The recordings and transcripts were examined by RM, IH and RB to identify talk about suicidal feelings and thoughts. The focus on suicidal ideation was motivated by the data because questions about suicidal behaviour were almost always preceded by questions about suicidal ideation. Typically, it was the case that only if the patient confirmed suicidal ideation, questions about suicidal behaviour followed. Psychiatrist/GP questions and patient responses about suicidal ideation were transcribed in detail using standardized conversation analytic methods to analyse the content of speech and speech delivery characteristics, such as pauses, overlap, stress, intonation, and pace [30].

Each question was analysed in terms of whether it was (1) open or closed and (2) negatively or positively framed. The response to each question was analysed. The psychiatric and primary care sample were analysed separately. Some patients volunteered information about feeling suicidal but no follow-up questions were asked. This occurred in 2.5% of visits.

#### Quantitative analyses

As previous work by Heritage et al. [31] has shown that doctors’ questions bias patients’ responses, the following hypothesis was tested: a negatively designed question is more likely to lead to a negative response. *χ* ^2^ was also used to explore whether patients with higher suicidal ideation were more likely to be asked a positively framed than a negatively framed question. Quantitative analyses were conducted on the psychiatric and primary care sample together.

## Results

Three hundred nineteen visits were screened. Suicidal ideation was assessed on 83 occasions by 35 psychiatrists in 77 visits (i.e., with 77 patients). In six visits, suicidal ideation was assessed twice. The number of patients per psychiatrist was 1-8. 70 (90.9%) patients had a diagnosis of schizophrenia and 7 (9.1%) had a diagnosis of depression. 61.4% were male. The mean patient age was 43.5 (SD 11.2, range 19-67). On the BDI or PANSS (available for 66 patients), 75.8% patients experienced less and 24.2% experienced more suicidal ideation.

**Fig. 1:**
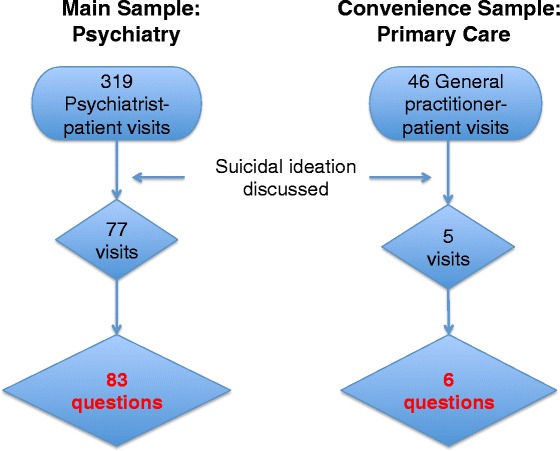
Flow Diagram of Psychiatric and Primary Care Sample

In primary care, suicidal ideation was assessed on 6 occasions in 5 visits (i.e., with 5 patients). In one visit, it was assessed twice. The mean patient age was 41 (SD 8.6, range 28-55). Four patients were male. The mean HADS score, available for 4 patients, was 15.3 (SD 3.2, range 13-21), indicating severe depression (Fig. 1).

There were three main findings.

### Psychiatrists tend to ask patients to confirm they are not suicidal

Psychiatrists initiated enquiries about suicide risk with a ‘gateway’ question. The response to this question determined further enquiry about risk or not. In all cases, this was a closed yes/no question. Closed questions can be answered with either a single word (yes or no) or a short phrase and are used to restrict the type of information received [31, 32]. For example, a typical question was “Do you ever feel that life is not worth living?” which invites a yes or a no response.

Of 83 questions, 62 (75%) communicated an expectation in favour of a no response and 21 (25%) communicated an expectation in favour of a yes response. In extract 1 below, the psychiatrist asks a preparatory question (line 1) “Not feeling low?”, a negative declarative statement, favouring a no response. The extracts below are conversation analytic transcripts. The transcription symbols are presented in Additional file 1. For reference, basic transcripts are presented in Table 1.

**Table 1:**
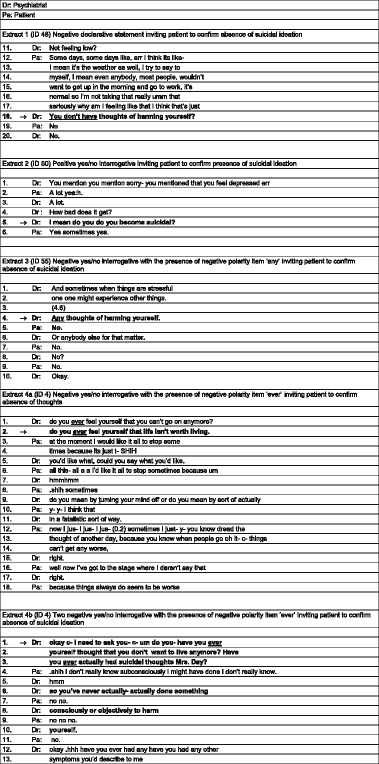
Basic transcription of extracts

In response to the question “Not feeling low?” the patient responds with a narrative conveying that she does feel low. The psychiatrist asks the gateway question as a negative declarative statement favouring a no response: “You don’t have thoughts of harming yourself?” (line 8). The patient responds with a ‘no’ (line 9).



By contrast, in extract 2, the, the gateway question (line 8) “I mean do you become suicidal” is positively framed with no negative polarity items. The patient responds “yes” without delay, which is qualified with “sometimes yes”.



In extract 3, the gateway question (line 4) is prefaced by a normalizing statement about when things are stressful. The question “Any thoughts of harming yourself” is negatively framed with the negative polarity item ‘any’. Note that this is not as strongly negatively framed as the negative declarative statement in extract 1.



The patient responds without delay with ‘no’ (line 5). The psychiatrist adds a second part to the question (line 6) “Or anybody else for that matter”. After a 0.8 s pause, the patient responds quietly with “No” (line 8). There is a checking “No?” (line 10) from the psychiatrist, followed by “okay”, displaying preparedness to move on to a new topic [33]. The risk assessment is complete and the talk moves on.

Doctors were not more likely to ask patients reporting more suicidal ideation a positively phrased than a negatively phrased question (*χ* ^2^ = 1.7, df = 1, p = 0.23).

Of the 6 primary care cases (see Table 2), 2 questions invited the patient to confirm that life was worth living. A further 2 questions were negatively framed communicating an expectation of no suicidal thoughts. The final 2 questions were positively framed communicating an expectation of suicidal thoughts. Hence, 4 of the 6 questions in primary care communicated an expectation of no suicidal ideation.

**Table 2:**
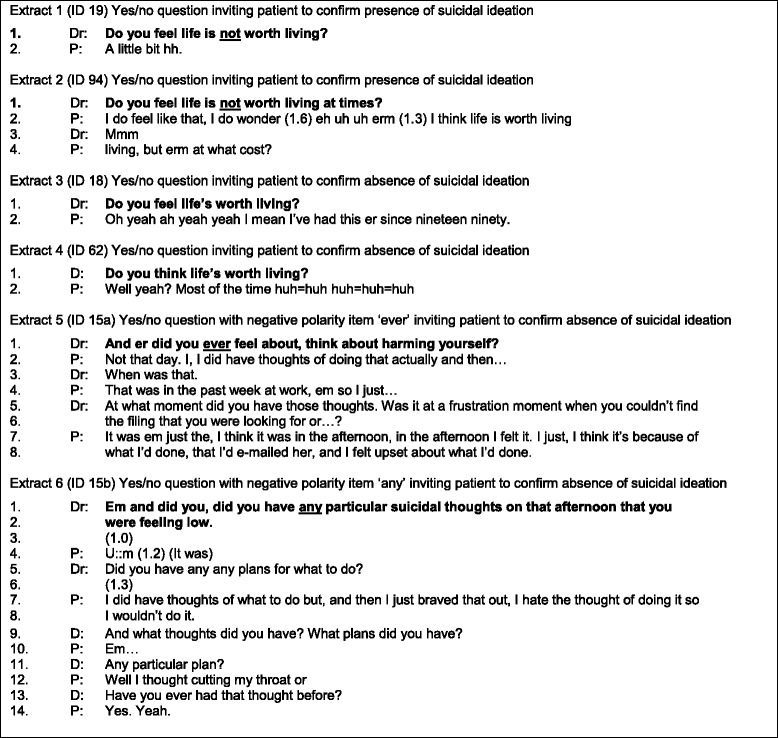
Six extracts from primary care

**Fig. 2:**
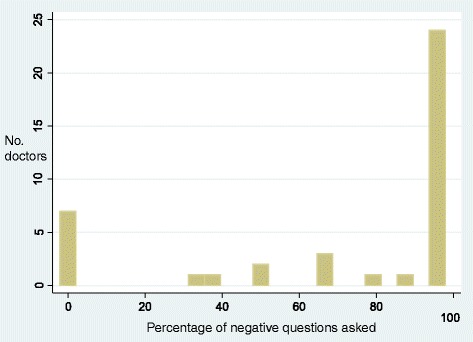
Distribution Of Questions Designed For No

How doctors used negatively and positively framed questions was examined (Fig. 2). Over half of the doctors (i.e., 24) always used negative framing while almost one-fifth (i.e., 7) always used positive framing.

### Patients were significantly more likely to say they were *not* suicidal when the question was negatively phrased

**Fig. 3:**
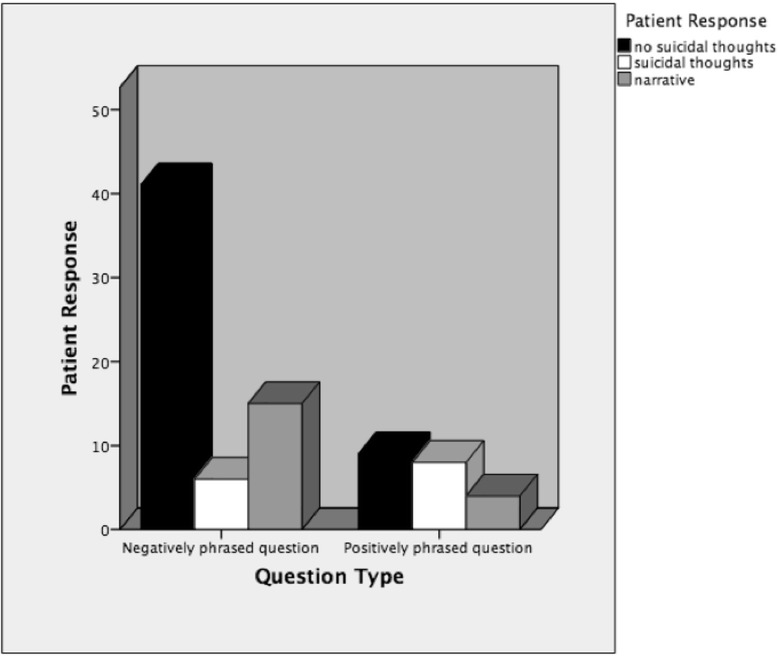
Distribution of Yes and No Responses by Question Type

When a doctor asks a patient a closed yes/no question, they place constraints on the kind of answer that should be provided, i.e., a yes or no [34]. As can be seen in Fig. 3, there were 62 negatively framed questions, which received 41 (66%) no responses, 6 (9.7%) yes responses and 15 (24.2%) narrative responses. Meanwhile, there were 21 positively framed questions, which received 9 (42.9%) no responses, 8 (38.1%) yes responses and 4 (19%) narrative responses.

When the question was negatively framed, patients were significantly more likely to say they were not suicidal but when the question was positively framed, patients were *not* more likely to say they were suicidal (*χ* ^2^ = 6.5, df = 1, *p* = 0.013).

The patient’s response to the gateway question was critical as it determined whether psychiatrists conducted further risk assessment. If patients responded no (in 60% cases), the talk moved on to other topics (e.g., extracts 3 and 6). If the patient responded yes, this led to detailed risk assessment.

### Patient narrative responses were pursued with closed yes/no questions

Yes/no questions invite brief responses. A yes or no response satisfies these constraints. However, a narrative response does not [32]. Patients responded with a narrative in around one-quarter of cases (19/83) indicating that the yes/no choice offered by the question was problematic. This is problematic for psychiatrists because it does not define risk in a clear-cut way. Hence, psychiatrists pursued a yes/no response following an initial narrative response.

In extract 4a, the psychiatrist poses a negatively framed question with the negative polarity item ‘ever’ (lines 1-2): “do you ever feel yourself that life isn’t worth living”. Following a micro-pause of less than 0.2 s, the psychiatrist reformulates the question (4-5), from “you can’t go on anymore” replacing it with a more specific description “life isn’t worth living”. The patient is crying and responds (6-7) with “at the moment I would like it all to stop sometimes because it’s just t-“. The psychiatrist seeks to clarify this utterance twice (9,11). The patient and psychiatrist are competing in overlapping talk (square brackets: 15-20) during which the psychiatrist (16) puts forward a possible understanding as “Do you mean by turning your mind off?”. The patient does not respond to this question and continues recounting her difficulties (lines 18-20). This continues for a further minute during which the patient is crying extensively.



The psychiatrist returns to the question in extract 4b (1-3), invoking a need to ask, thereby minimizing her agency in asking the question: “I need to ask you-“. Again the question is ‘no inviting’ and modified from line 4, extract 4a. This version “do you- have you ever thought .hhh that you don’t want to live anymore?” is quickly reformulated to “Have you ever actually had suicidal thoughts Mrs. Day?”, another ‘no inviting’ question, upgraded from “don’t want to live anymore” to “suicidal thoughts”. The patient responds again with a narrative (4-5), hedging her response “I don’t really know…”.



Following two unsuccessful attempts to secure a yes/no, the psychiatrist moves from thoughts to behaviour and seeks confirmation that the patient has not done something to harm herself (7-11) with another closed negatively framed declarative question “so you’ve never actually done something consciously or objectively to harm yourself”. Once a ‘no’ response is secured (8,10,12), the psychiatrist receipts with “okay” and moves on to other symptoms. As with the question about suicidal ideation, the question about suicidal behaviour is a negatively phrased question inviting the patient to confirm that she has not acted to harm herself.

## Discussion

There were three main findings from this study. Firstly, questions about suicidal ideation were closed yes/no questions designed to constrain the patient’s response to a yes/no. All were leading questions with three-quarters inviting the patient to confirm they were not feeling suicidal. More than half of the psychiatrists always framed the question negatively, with a minority always framing the question positively. Secondly, a subtle difference in the wording of the question biased the patient’s response. Negatively framed questions significantly biased the patient’s response towards a no ‘suicidal ideation’ response. If the patient responded yes, further information gathering was conducted. However, if the patient responded no, the psychiatrist moved on to other topics with no further risk assessment.

Finally, patients responded with a narrative in one-quarter of cases. Narratives conveyed some suicidal thoughts and were pursued with closed yes/no questions.

That questions about suicidal ideation were more likely to be negatively framed is consistent with other research on doctor questioning. Typically, doctors design questions for the ‘best case’ patient outcome, e.g., “Not feeling low?”, identified as the principle of optimization, a default feature of medical questions [25]. Previous research also found that doctors’ questions bias patients’ responses. In a randomised controlled trial, doctors who asked “Do you have *some* other concerns you would like to discuss?” inviting a yes, versus “Do you have *any* other concerns you would like to discuss?”, inviting a no, were significantly more likely to elicit and reduce unmet concerns compared before and after the visit [31].

In asking about suicidal ideation, optimized or ‘no problem’ questions are problematic because they minimise the disclosure of suicidal ideation, a tension also described in other medical settings [35]^.^ Gao et al. [36] found that patients were more likely to minimize the frequency and severity of suicidal ideation during clinician ascertained assessment compared to self-report. The current study sheds some light on these and other findings from the U.K. National Confidential Inquiry into Suicide [37] that most people who took their life were classified as ‘low risk’ in contacts with mental health services. In the U.S., Smith et al. [38] also found that most patients dying by suicide “denied suicidal ideation” in their final contact with services. Furthermore, Haynal-Reymond et al. [39] found that psychiatrists’ written predictions predicted 22.7% of future attempts. However, psychiatrists’ nonverbal behaviour, specifically frowning and gazing at the patient for longer, predicted around 90% of future attempts. This suggests a perception of risk, of which doctors are not consciously aware, that is overridden by verbal communication.

The findings should be considered in light of the study’s strengths and limitations. This is the first systematic analysis of how psychiatrists interview patients about suicidal ideation using real time data. Conversation analysis shows how one word can tilt the question positively or negatively. Although the findings were similar over time across different psychiatric samples, they may be specific to these patient groups and settings. The data, although collected across urban, semi-urban and rural settings, were collected in the U.K and practice may vary across countries. This qualitative study did not study factors such as diagnosis, sex and previous suicide attempts. A gold standard assessment such as the Columbia Suicide Severity Rating Scale was not used. However, such scales are not used in routine practice. Nevertheless, psychiatrists’ choice of questioning may reflect their intuitive assessment of risk. Finally, the consent rate was less than 50%: patients who consented may not be fully representative of the patient population.

There are various reasons why psychiatrists may use negatively framed questions. They will be aware of the workload implications of a yes response, i.e., the need for a more in depth assessment and potentially onward referrals. They may also believe that more extensive assessment and escalation of bureaucratic procedures is not in the patient’s best interest. Moreover, patients and psychiatrists may collude in not talking about suicide because it is emotionally difficult for everyone.

The findings suggest a dilemma for around one-quarter of patients who responded with a narrative conveying ambivalence. If they say no, despite some suicidal thoughts their care could be compromised. However, conveying a clear yes could result in a less welcome response: further intrusive questions, closer observation, a formal mental health assessment or the possibility that their children may be removed from their care. For patients, problems that are difficult to put into words or of questionable legitimacy tend to be presented in narratives [40]. For psychiatrists, narratives are also problematic as it is more difficult to classify risk in a categorical way.

These findings have implications for clinical practice. Positively framed questions do not bias the patient’s response as negatively framed questions do. As recommended in U.S. [41] and U.K. [42] suicide Prevention Strategies, professionals could benefit from training in eliciting suicidal ideation.

## Conclusions

Psychiatrists ask patients closed yes/no questions about suicidal ideation. It is not possible to ask a non-leading, closed question. The majority of questions communicated an expectation in favour of a ‘no’ response. These questions biased patients’ responses towards reporting no suicidal ideation. Hence, they may not be optimal in eliciting suicidal ideation.

## Additional file

Additional file 1: Transcription conventions. Transcription Conventions. Explanation of transcription symbols used in analysing the communication data. (DOCX 42 kb)
