# Supplementary material for: How do healthcare professionals interview patients to assess suicide risk?
Source: BMC Psychiatry. 2017 Apr 4;17:122. doi: 10.1186/s12888-017-1212-7 (PMC5379679; doi:10.1186/s12888-017-1212-7)
Supplement: Additional file 1: — Transcription conventions. Transcription Conventions. Explanation of transcription symbols used in analysing the communication data. (DOCX 42 kb) [file 12888_2017_1212_MOESM1_ESM.docx]

**Transcription conventions**

.hhh Audible inhalation

hhh Audible exhalation

: Extended sound

↑ Rising intonation

↓ Falling intonation

? Rising inflection

____ Emphasis (word or part of word underlined)

° ° Talk is quieter than the surrounding talk

< > Talk is faster than the surrounding talk

UPPERCASE Talk is louder than the surrounding talk

! Animated tone

= Latched utterance, no interval between utterances

[ ] Beginning and end of overlapping talk

( ) Transcriptionist doubt

(.) A pause of less than 0.2 seconds

1. Silence measured in seconds and tenths of seconds
